# Teacher or Artificial Intelligence? The Effect of Decision-Making Agent on Junior High School Students’ Decision Acceptance: A Moderated Mediation Model

**DOI:** 10.3390/bs16071227

**Published:** 2026-07-19

**Authors:** Zhen Zhang, Siyu Fan, Chunhui Qi

**Affiliations:** 1 Faculty of Education, Henan Normal University, Xinxiang 453007, China; zhangzhen201912@htu.edu.cn (Z.Z.); 2410283130@stu.htu.edu.cn (S.F.); 2 Faculty of Education, Henan University, Kaifeng 475001, China

**Keywords:** artificial intelligence, teacher decision-making, decision acceptance, perceived fairness, explanatory feedback, moderated mediation

## Abstract

Artificial intelligence (AI) is increasingly used in educational decision-making. This raises important questions about how students perceive and accept outcomes from non-human agents. Based on authority acceptance theory and organizational justice theory, this study explored three research questions: how decision-making agents (teachers vs. AI) affect junior high school students’ acceptance of educational decisions, the mediating mechanism of perceived fairness, and the moderating effect of explanatory feedback. This study adopted a 2 (decision-making agent: teacher vs. AI) × 2 (explanatory feedback: with vs. without explanation) between-subjects experimental design, with 250 seventh-grade students as participants. Results showed that students reported significantly higher decision acceptance and perceived fairness for teacher-made decisions than for AI-generated decisions. Perceived fairness partially mediated the relationship between the decision-making agent and decision acceptance. The mediating effect accounted for 67.52% of the total effect. Explanatory feedback positively moderated only the path from the decision-making agent to perceived fairness, with no significant moderating effect on the direct link between the decision-making agent and decision acceptance. Provision of explanations widened the human–AI gap in perceived fairness, which in turn further enlarged the gap in decision acceptance via the mediating pathway of perceived fairness. These findings extend research on explanatory feedback to the basic education context, while highlighting the significance of teacher-led human–AI collaboration and interpretable AI design amid the digital transformation of education.

## 1. Introduction

With the continuous iteration and advancement of big data and artificial intelligence technologies, the digital transformation of education has evolved into an irreversible developmental trend. In 2024, UNESCO released Six Pillars of the Digital Transformation of Education: A General Framework to steer the global digital shift in inclusive education, highlighting that equity has not been prioritized sufficiently in worldwide educational digitalization practices. As educational decision-making is transforming from experience-driven governance toward human–AI collaborative governance, decision-making agents (human teachers vs. artificial intelligence) emerge as a core determinant governing the implementation of educational policies and students’ developmental outcomes. Differences in decisions generated by distinct decision-making agents and students’ resultant acceptance attitudes have therefore become a critical research priority across multiple educational contexts, including mental health instruction, academic performance assessment and student behavioral management (Du & Lv, 2024; Zhang & Aslan, 2021; Mouta et al., 2024).

In contemporary organizations, decision-making is no longer exclusively undertaken by traditional human decision-makers such as managers and supervisors, but is also performed by artificial intelligence (AI; Choi & Chao, 2024). For instance, AI systems are increasingly deployed to fulfill organizational decision-making tasks ranging from personnel recruitment and promotion to risk evaluation and attendance monitoring (Jones-Jang et al., 2025), bringing about a fundamental restructuring of the constituent bodies for organizational decision-making.

Leveraging efficient data algorithms and standardized evaluation criteria, artificial intelligence systems have gradually evolved into emerging decision-making agents within education (Popenici & Kerr, 2017). The infiltration of AI algorithms into educational practices has become inevitable, covering the assessment of students’ academic proficiency, personality and mental health, as well as rewards and disciplinary sanctions for daily behavioral norms (Chai et al., 2024). For example, researchers have built models based on natural language processing to spot grammatical mistakes and identify argumentation frameworks, enabling automatic and precise essay scoring (Fu et al., 2018). In addition, machine learning has facilitated the development of an automated classroom assessment system capable of observing and documenting teacher–student interactions alongside real-time feedback delivery (James et al., 2018). Unlike teachers whose decisions rest on accumulated teaching experience, subjective perceptions and personal affective inclinations (Wang, 2021), AI-driven decisions feature standardized criteria, consistent outcomes, full traceability and freedom from subjective bias (Holmes et al., 2021; Owan et al., 2023). Nevertheless, educational settings are inherently complex. On the one hand, as direct recipients of educational decisions, students’ willingness to accept outcomes hinges not merely on assessment accuracy but also on psychological factors such as emotional identification, humanistic perception and interpersonal trust (Wang, 2021; Mouta et al., 2024). Sustained in-school interactions help foster stable emotional bonds and mutual trust between teachers and students. Accordingly, teachers’ decisions that strike a balance between formal institutional rules and humanistic care tend to gain greater recognition from students (Van Maele & Van Houtte, 2011). By contrast, prominent drawbacks, including insufficient affective empathy and algorithmic black boxes, greatly undermine students’ acceptance of AI rulings (Holmes et al., 2021). On the other hand, educational decisions originate from authentic classroom contexts. Teachers access abundant contextual information through face-to-face communication with students when making judgments (Shadiev et al., 2017; Archambault et al., 2022), whereas AI decision-makers are prone to decoupling from practical educational scenarios and struggle to adapt feedback through real-time student engagement, resulting in decisions failing to reconcile practical requirements with learners’ emotional needs (Littman, 2015; Nguyen Thanh et al., 2023).

In summary, teachers and AI have become the two dominant decision-making agents in contemporary educational contexts, yet the existing literature bears notable limitations. For one thing, prior research overwhelmingly centers on comparisons of technical efficiency and scoring accuracy across different decision-makers, while overlooking discrepancies in students’ decision acceptance and humanistic attributes induced by agent types. For another, few dedicated empirical studies take the decision-maker category (teacher versus AI) as the core independent variable to examine students’ attitudinal responses toward resultant decisions. Most relevant empirical works prioritize technical performance rather than students’ subjective psychological perceptions, leaving the divergent impacts of decision-making agents on acceptance unexamined and with insufficient empirical evidence to corroborate the core hypotheses of the present research.

### 1.1. The Influence of Decision-Making Agent on Decision Acceptance

Artificial intelligence develops rapidly. It has evolved from basic automation tools into collaborative intelligent agents. Unlike traditional IT systems, modern AI is no longer a passive tool for human use or fully controlled by humans. It can take on task responsibilities and pursue optimal solutions amid uncertainty (Baird & Maruping, 2021), gradually emerging as an independent decision-making agent. Baird and Maruping (2021) proposed a theoretical framework for such agentic information systems. Their core feature lies in autonomous goal-setting, cross-party interaction with humans or other machines, and dynamic adaptation to shifting environments. Accordingly, human–AI relations shift from the conventional user-tool mode to complex principal-agent ties. Tasks, rights and responsibilities are no longer assigned unilaterally but dynamically negotiated and distributed by both sides (Baird & Maruping, 2021). Existing research confirms that AI agents are widely viewed as formal team members (Dennis et al., 2023). This change brings benefits such as fewer conflicts during high-performance work. Meanwhile, it creates new troubles, including lower procedural satisfaction and weakened perceived benevolence. Such subtle shifts in AI’s identity restructure power and authority within organizations. When AI evolves from executing human instructions to participating in independent decision-making and assuming corresponding responsibilities, the ownership and legitimacy of decision-making authority become an urgent research topic.

Based on the acceptance-of-authority theory proposed by Barnard (1968), authority exists in issued orders. An order gains legitimate authority only when recipients acknowledge and implement it. In education, teachers and educational institutions have long served as the dominant decision-makers. As traditional authoritative bodies, they release educational decisions to guide student development. Students grow accustomed to their evaluative standards and decision-making status. Against this backdrop, AI emerges as a new decision-maker in educational settings. It replaces the conventional authoritative role of teachers. This raises a core question: can students accept decisions generated by AI instead of traditional human authorities?

Past research finds that human decision-makers (HDMs) produce higher acceptance of decisions than algorithmic decision-makers (ADMs) (Wesche et al., 2024). Owing to public skepticism of AI agents, pervasive distrust and negative perceptions prevail regarding AI-derived decisions. This bias stands out for judgments involving human traits, such as subjective evaluation and personal affection (Bonaccio & Dalal, 2006; Daschner & Obermaier, 2022; Vanneste & Puranam, 2025). In addition, algorithms are seen as less fair and credible than human judges (Lee, 2018). This reduces people’s acceptance and behavioral compliance (Geiskkovitch et al., 2016). For instance, many studies note unfavorable user responses toward algorithmic decisions in workplace scenarios (Langer & Landers, 2021; Parent-Rocheleau & Parker, 2021). Researchers trace this tendency to perceived algorithmic black boxes and stricter expectations for explanatory feedback (Zerilli et al., 2018). Accordingly, the first research hypothesis is proposed as follows:

**H1.**  *Students exhibit higher acceptance toward decisions made by teachers than those generated by AI decision-makers.*

### 1.2. Perceived Fairness as a Potential Mediator Variable

Fairness is a core human value and a fundamental rule to sustain social order. Based on organizational justice theory, Colquitt (2001) defines fairness as people’s perceived compliance with proper rules in decision-making contexts. Prior studies show that people form different fairness perceptions toward identical decisions. Such differences depend on who makes the decision (Choi, 2008; Hollensbe et al., 2008). This pattern is especially obvious when comparing human and AI decision-making. It has become a key research topic in human–machine interaction and collaboration.

Existing research has mixed findings about how decision-makers shape perceived fairness. On the one hand, empirical evidence suggests people hold higher fairness and understandability perceptions for human decisions than for AI decisions. Shulner-Tal et al. (2025) ran a between-subject online experiment. Their results show participants report significantly lower fairness and comprehensibility for AI-generated decisions. Simply knowing a decision comes from AI alone hurts perceived fairness. A relevant study focuses on undergraduate essay grading and feedback in higher education. Even when students cannot reliably tell who graded their work, human raters bring more favorable subjective feelings. Once students learn scores are from AI, their confidence in grading outcomes drops noticeably (Thomas et al., 2025). However, other studies draw opposite conclusions. Choi and Chao (2024) conducted six experiments with a total of 2794 participants. They found that when decisions produce unfavorable outcomes for individuals, AI decisions are rated as fairer than human decisions. People also respond less negatively to disappointing results from algorithms. The underlying reason is that AI is viewed as emotion-free and thus more objective and impartial. In addition, research set in extremely unfair contexts proves that higher perceived fairness boosts people’s trust in decision-makers. Improved trust further raises their willingness to interact with the same decision-makers later (Shin & Lee, 2025). These inconsistent results reveal that the association between decision source and perceived fairness is not unidirectional; it is jointly shaped by outcome favorability, contextual background, and individuals’ pre-existing attitudes toward AI.

After clarifying the relationship between decision-makers and perceived fairness, it is necessary to further explore how fairness perception affects individuals’ acceptance of decisions. Existing research confirms that people who perceive a decision process as fair tend to adjust their behaviors and accept corresponding outcomes (Brockner et al., 2001). Choi and Chao (2024) directly measured decision acceptance as a downstream outcome of perceived fairness. They argued that a greater perception of fairness raises decision acceptance and future engagement willingness. With the expanding use of AI decisions in organizational evaluation, this causal chain gains more empirical support. Laboratory experiments by Shin and Lee (2025) found that perceived fairness improves trust and positively promotes users’ adoption intention. Their findings provide process evidence for the behavioral transformation of fairness perception. In the educational field, some scholars construct an AI-mediated educational decision model grounded in organizational justice theory. The model proposes that students’ perceived fairness sequentially predicts their acceptance of specific decisions and overall trust in educational institutions (Turner, 2025). Moreover, prior work demonstrates that perceived fairness effectively mitigates students’ resistance against AI evaluation systems (Jones-Jang et al., 2025). Accordingly, perceived fairness is more than a simple psychological or emotional experience; it acts as a core mediator linking decision-maker characteristics and final decision acceptance. On these grounds, Hypothesis 2 is proposed.

**H2.**  *Perceived fairness mediates the association between decision-making agents and decision acceptance; specifically, decision-making agents predict students’ decision acceptance indirectly via perceived fairness.*

### 1.3. Explanatory Feedback as a Potential Moderator

Explanatory feedback acts as a critical contextual factor shaping individuals’ perceived fairness and decision acceptance. Ellis et al. (2009) defined informational justice as the extent to which decision-makers provide adequate justifications for decisions and procedures. Through sufficient information communication and explanation, recipients can understand the underlying logic and process of decisions. Previous studies have shown that adequate explanations help employees feel fair within the team and enhance their acceptance and cooperation intentions (Colquitt, 2001; Zheng & Liu, 2016). On the contrary, insufficient information provided will fuel rumors and distort individuals’ emotions and behaviors (Citera & Rentsch, 1999. When decision-making approaches are consistent, accurate, impartial, reasonable and transparent, recipients tend to develop strong perceptions of fairness (Rasooli et al., 2019).

**Figure 1:**
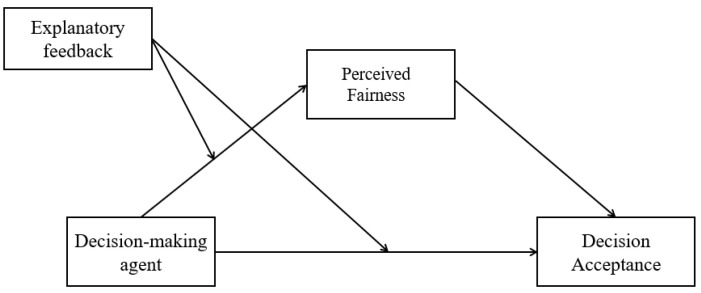
Research model.

In educational contexts, students’ perceptions regarding the fairness of decision outcomes are largely shaped by the explanatory feedback they receive (Hacker et al., 2008). When such feedback fails to deliver a sense of fairness to students, it will further reduce their satisfaction with decision outcomes, acceptance of the decisions, and future behavioral intentions (Kizilcec, 2016). More importantly, explanatory feedback may moderate the effect of the decision-making agent (human vs. AI) on perceived fairness and decision acceptance. In contexts with low explanatory feedback, ambiguous AI outputs and the absence of explanations for outcomes exacerbate algorithmic bias and widen the acceptance gap between human and artificial intelligence decisions (De Bruijn et al., 2022). In contrast, high explanatory feedback with adequate explanations and information disclosure offsets AI’s trust deficit and brings its perceived fairness and acceptance close to human-made decisions. For instance, Grimmelikhuijsen (2023) found that AI decisions gained markedly lower acceptance than human decisions under low explanatory feedback, whereas such gaps disappeared or even reversed under high explanatory feedback. Wesche et al. (2024) reached consistent findings in recruitment settings: increasing the level of explanatory feedback can significantly narrow the acceptance gap between human decisions and algorithmic decisions. In summary, the hypothetical model proposed in this study is shown in Figure 1.

**H3.**  *Explanatory feedback positively moderates the relationship between decision-making agent and perceived fairness, as well as between decision-maker agent and decision acceptance.*

## 2. Methods

### 2.1. Participants

Before formal hypothesis testing, GPower 3.1 was used for a priori power analysis to calculate the minimum sample size for the four-group between-subjects ANOVA (main effects and interaction effects). Consistent with existing educational research on decision acceptance and fairness perception, we set the medium effect size *f* = 0.25, *α* = 0.05, target statistical power 1 − *β* = 0.85, numerator *df* = 1, and four experimental groups. The simulation output noncentrality parameter *λ* = 9.12, critical *F* = 3.90, denominator *df* = 142, and the minimum valid sample size *N* = 146 to effectively detect the hypothesized main and interaction effects. Sample size was calculated in advance via GPower 3.1, which indicated a minimum sample of 146 participants to achieve a statistical power of 0.85, and a total of 258 seventh-grade students were recruited from a junior high school in a city of Henan Province, China, notably, statistical power varies substantially across different pathways within the full moderated mediation model, as a sample size sufficient to identify simple main factor effects may fail to provide adequate power to detect weaker interaction terms or the index of moderated mediation given that conditional indirect and interactive effects generally produce smaller population effect sizes than main effects, and our final valid analytical sample of 250 participants retained after data screening far exceeds the power-derived minimum threshold of *N* = 146, which guarantees satisfactory statistical power to test all core hypotheses covering group main effects, two-way interaction effects, and the moderated mediation index in the structural model.

Three exclusion criteria were predefined before data collection to filter invalid responses: participants who failed manipulation check questions for decision agent or explanatory feedback priming, and participants who provided identical answers to all scale items were excluded from the final analytical sample. In total, 8 student participants were excluded from the dataset according to these standards, yielding a final valid sample of 250 students (126 boys, 50.40%; 124 girls, 49.60%) with a mean age of 13.27 years.

A detailed breakdown of excluded participants across experimental conditions is provided as follows. Five participants failed the manipulation check measuring recognition of the decision-maker role: 2 from the teacher-decision with explanatory feedback group, 2 from the AI-decision with explanatory feedback group, and 1 from the AI-decision without explanatory feedback group. An additional 3 participants were eliminated for submitting invariant identical responses to all questionnaire items, while no invalid responses were detected in the teacher-decision without explanatory feedback group. All manipulation check outcomes for the decision agent manipulation are fully reported herein. Manipulation check items distinguishing between the two decision agent types (teacher vs. AI) were administered immediately prior to the measurement of the dependent variables, namely perceived fairness and decision acceptance.

### 2.2. Experimental Design

A 2 (decision-making agent: AI vs. teacher) × 2 (explanatory feedback: explanation vs. without explanation) between-subjects experimental design was adopted. Referring to the homework grading paradigm from Jones-Jang et al. (2025), this study constructed a scenario concerning the evaluation of regular seventh-grade mathematics exercises rated as medium difficulty by subject teachers. After reading the experimental vignette, participants finished manipulation checks before completing paper-and-pencil scales measuring perceived fairness and decision acceptance.

This research employed cluster randomization with intact classes as the unit of random assignment. Four complete student classes were randomly allocated to four separate experimental conditions through random number generation. Specifically, each class was assigned one unique experimental scenario by random draw, with identical allocation probability for all groups. In terms of sample characteristics, the four classes shared similar demographic compositions and equivalent pre-experiment attitudinal baselines. All experimental sessions were carried out under unified administrative management and standardized classroom environments, including consistent experimental materials, operating instructions and on-site supervision criteria to control extraneous variables. Notably, random assignment was executed at the class level rather than the individual level. All students within a single class received the same experimental treatment; no students from the same class were assigned to different experimental environments during the study.

All data collection was conducted on a voluntary and anonymous basis to mitigate expectancy and response biases. Participants were informed that all collected data would be used solely for academic research without any disclosure of personal, identifiable information, and there were no right or wrong answers. Before the experiment began, we obtained the consent of the students and their parents, and the students themselves signed the informed consent form.

#### 2.2.1. Control Variables

To control for individual discrepancies, participants were asked to report their age, gender, as well as their frequency and familiarity with AI usage.

#### 2.2.2. Procedures and Manipulations

Participants were randomly assigned to one of four experimental conditions formed by the 2 (teacher vs. AI) × 2 (explanation vs. without explanation) factorial design. All participants read an identical basic vignette describing a mathematics homework grading event, with only two manipulated factors varied across groups: the source of the grading decision (teacher or AI) and explanatory feedback (with or without explanatory feedback). The core scenario was presented as follows:

*You finished a set of mathematics exercises yesterday. Your work has been graded out of 10 points today, and the score will be archived to track your academic progress. All students’ assignments are scored either by an automated AI grading system or by your regular math teacher based on the correctness of solutions and final answers.*

*The AI system (or math teacher) provided your score accompanied by grading feedback: “Points deducted for skipped computational steps and missing key derivations, resulting in a 4-point penalty.” (No explanatory feedback was provided in the no-explanation condition), leaving your final score at 6 out of 10.*

Following vignette exposure, manipulation checks were administered to verify experimental manipulations. One item assessed the effectiveness of the explanatory feedback manipulation by asking participants to rate the level of explanatory information provided in the scenario on a 5-point Likert scale, with higher scores indicating greater perceived explanatory feedback. A separate single-item check validated the decision-maker manipulation: “Who graded your homework in the scenario? 1 = AI, 2 = Teacher, 3 = Cannot remember”.

### 2.3. Measures

#### 2.3.1. Measurement of Perceived Fairness

Perceived fairness was measured using the two-item scale developed by Jones-Jang et al. (2025) with a 5-point Likert response format. Participants rated two statements: (A) whether the evaluation by the teacher (or AI) was fair, and (B) whether their assigned score was reasonable. The Cronbach’s α coefficient for this scale was 0.90; higher aggregate scores represented stronger perceived fairness.

#### 2.3.2. Measurement of Decision Acceptance

Acceptance of the decision was measured using the four-item scale developed and adopted by Yang et al. (2026). Sample items included “I accept the grading result given by the AI system” (see Appendix A). Responses were rated on a 7-point Likert scale ranging from 1 (strongly disagree) to 7 (strongly agree). The Cronbach’s α of the scale was 0.89, with higher total scores indicating greater decision acceptance.

All measurement indicators were translated, culturally revised and adapted to fit the cognitive level and school context of seventh-grade Chinese students. Original scale items were first translated into simplified Chinese and rephrased to match the daily learning scenarios and comprehension abilities of junior high school adolescents. Next, five postgraduate and doctoral researchers specializing in educational psychology and pedagogy reviewed all items, providing revision suggestions on wording and expression to optimize semantic accuracy. Prior to formal data collection, five students from each participating class read through all questionnaire items in advance under the guidance of their head teachers, and no feedback regarding confusing or incomprehensible statements was received, which preliminarily verified the age appropriateness of the adapted scale. To eliminate wording bias between experimental conditions, we maintained fully equivalent sentence structures, length, descriptive detail and neutral tone for all introductory instructions and experimental priming materials that described the two decision agent types (teachers and AI systems) across all four experimental questionnaires. Furthermore, we conducted factor analysis validity tests on the four core scale items; the Kaiser-Meyer-Olkin (KMO) measure of sampling adequacy reached 0.82, and Bartlett’s test of sphericity yielded an approximate chi-square value of 602.31 with *df* = 6 at *p* < 0.001. These statistical results confirmed that the dataset was suitable for factor analysis and provided solid quantitative evidence for the construct validity of the adapted measurement instrument.

## 3. Results

### 3.1. Manipulation Checks

One single-item manipulation check was used to verify the experimental manipulation of explanatory feedback. Results confirmed a successful manipulation of explanatory information provision: participants in the explanation-present condition (*n* = 127) reported significantly higher scores on the manipulation check item (*M* = 3.37, *SD* = 0.83) relative to those in the without explanation condition (*n* = 123, *M* = 2.08, *SD* = 0.98), one-way ANOVA yielded *F*(1,248) = 124.46, *p* < 0.001, with the total valid sample size of the analysis being *N* = 250.

### 3.2. Preliminary Analyses

Correlation analyses were conducted among acceptance of decision, decision-making agent, perception of fairness, and explanatory feedback (see Table 1). Perceived fairness, explanatory feedback, and decision acceptance were significantly positively correlated with one another. The decision-making agent was positively correlated with decision acceptance (r = 0.35 **), which preliminarily supported Hypothesis 1.

A 2 × 2 factorial ANOVA was performed with perceived fairness as the dependent variable. The main effect of the decision-making agent was significant, *F*(1,246) = 25.85, *p* < 0.001, partial *η*^2^ = 0.095. Participants reported significantly higher perceived fairness under the teacher condition (*M* = 3.58, *SD* = 1.01) than under the AI condition (*M* = 2.99, *SD* = 0.93), *p* < 0.001. The main effect of explanatory feedback was also significant, *F*(1,246) = 20.90, *p* < 0.001, partial *η*^2^ = 0.07. Perceived fairness was markedly higher in the explanation-provided group (*M* = 3.55, *SD* = 0.97) relative to the no-explanation group (*M* = 3.02, *SD* = 0.99), *p* < 0.001. Moreover, the two-way interaction between the decision-making agent and explanatory feedback significantly predicted perceived fairness, *F*(1,246) = 5.99, *p* = 0.015, partial *η*^2^ = 0.02. A follow-up simple-effect analyses indicated that, for both decision-maker conditions, perceived fairness was significantly greater when grading explanations were available: Teacher condition: with explanation (*M* = 3.99, *SD* = 0.79) > without explanation (*M* = 3.17, *SD* = 1.03); AI condition: with explanation (*M* = 3.11, *SD* = 0.92) > without explanation (*M* = 2.86, *SD* = 0.92).

**Figure 2:**
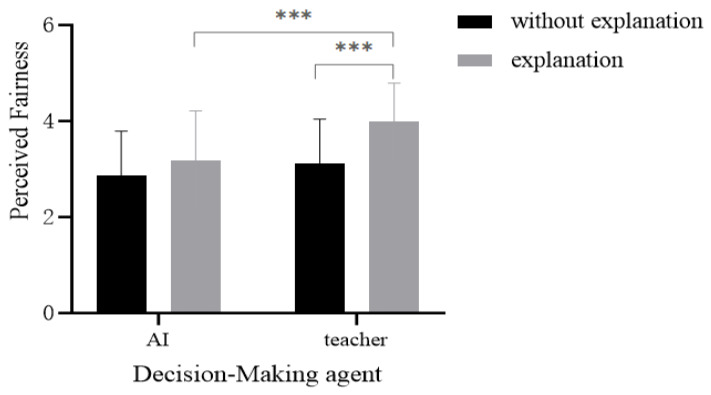
Effects of decision-making agent on perceived fairness. *** indicate significance at the 0.001.

A 2 × 2 factorial ANOVA was conducted with decision acceptance as the dependent variable. The main effect of the decision-making agent was statistically significant, *F*(1,246) = 40.61, *p* < 0.001, partial *η*^2^ = 0.14. Participants exhibited significantly higher decision acceptance in the teacher condition (*M* = 4.48, *SD* = 1.50) compared with the AI condition (*M* = 3.47, *SD* = 1.16), *p* < 0.001. A significant main effect of explanatory feedback was also observed, *F*(1,246) = 32.01, *p* < 0.001, partial *η*^2^ = 0.11. Decision acceptance was significantly higher for participants receiving explanatory feedback (*M* = 4.42, *SD* = 1.41) than for those without explanations (*M* = 3.52, *SD* = 1.30), *p* < 0.001. By contrast, the interaction between the decision-making agent and explanatory feedback failed to reach significance for decision acceptance, *F*(1,246) = 1.08, *p* = 0.30, partial *η^2^*= 0.004 (see Table 2 and Figure 2).

### 3.3. Moderated Mediation Model

The moderated mediation model was tested via the PROCESS macro (Model 8) embedded in SPSS 26.0. In this model, the decision-making agent served as the independent variable, decision acceptance as the dependent variable, perceived fairness as the mediator, and explanatory feedback as the moderator. Given the binary nature of the decision-making agent and explanatory feedback, dummy coding was implemented prior to analysis: AI = 0, teacher = 1; explanation = 1, without explanation = 0. All continuous variables were standardized, and participants’ familiarity with AI and frequency of AI usage were included as covariates in the model.

Results are presented in Table 3. In the regression equation predicting perceived fairness, the overall model fit was satisfactory (*R*^2^ = 0.17, *F* = 13.27, *p* < 0.001). The decision-making agent significantly and positively predicted perceived fairness (*β* = 0.88, *t* = 5.38, *p* < 0.001, 95% CI [0.56, 1.21], excluding zero). The predictability of explanatory feedback on the perception of fairness is not significant (*β* = 0.24, *t* = 1.43, *p* > 0.05, 95% CI [−0.08, 0.56], containing zero). There was a significant positive interaction between the decision-making agent and explanatory feedback (*β* = 0.58, *t* = 2.45, *p* < 0.05, 95% CI [0.11, 1.03], excluding zero), indicating that explanatory feedback attenuates the positive predictive effect of the decision-making agent on perceived fairness.

In the regression model predicting decision acceptance, the overall model exhibited good fit (*R*^2^ = 0.57, *F* = 51.60, *p* < 0.001). The decision-making agent significantly and positively predicted decision acceptance (*β* = 0.58, *t* = 3.24, *p* < 0.01, 95% CI [0.24, 0.95], excluding zero). Perceived fairness is positively associated with decision acceptance and serves as a correlational mediator in the model. (*β* = 0.90, *t* = 13.74, *p* < 0.001, 95% CI [0.77, 1.02], excluding zero). Explanatory feedback also positively and significantly predicted decision acceptance (*β* = 0.52, *t* = 3.07, *p* < 0.01, 95% CI [0.19, 0.86], excluding zero). By contrast, the interaction term between the decision-making agent and explanatory feedback failed to significantly predict decision acceptance (*β* = 0.19, *t* = 0.77, *p* > 0.05, 95% CI [−0.29, 0.66], containing zero).

Table 4 reports the direct and indirect mediating effects under distinct values of the moderator variable. For the group with explanatory feedback, the direct effect was 0.38 with a 95% bootstrap confidence interval of [0.30, 0.73], which excluded zero. For the group without explanatory feedback, the direct effect was 0.57 with a 95% bootstrap confidence interval of [0.23, 0.90], which also excluded zero. Regarding indirect effects, the indirect effect of the explanatory group was 0.79, with a 95% bootstrap confidence interval of [0.52, 1.09] that excluded zero. By contrast, the indirect effect of the non-explanatory group was 0.27, with a 95% bootstrap confidence interval of [−0.03, 0.59] that contained zero, indicating a non-significant indirect effect for this subgroup. Both subgroups yielded significant direct effects, yet only the explanatory subgroup produced a significant indirect effect. We further tested the overall moderated mediation effect via the index of moderated mediation (Index). The results showed that the index value was 0.51, with a 95% bootstrap confidence interval of [0.12, 0.91], excluding zero, which verified a statistically significant overall moderated mediation effect. In short, explanatory feedback exerted a stronger direct impact in the absence of explanatory narratives; only when supporting explanations were provided could explanatory feedback exert a significant indirect influence through the mediating pathway.

Bootstrap sampling with 5000 resamples was adopted to test the indirect effect, and an effect was deemed significant if its 95% confidence interval excluded zero. Results revealed a significant partial mediating effect of perceived fairness between the decision-making agent and decision acceptance. The indirect effect (ab = 0.79), direct effect (c′ = 0.38), and total effect (ab + c′ = 1.17) indicated the mediating pathway of perceived fairness explained 67.52% of the overall relationship between the decision-making agent and decision acceptance. Accordingly, Hypothesis 2 was supported.

**Figure 3:**
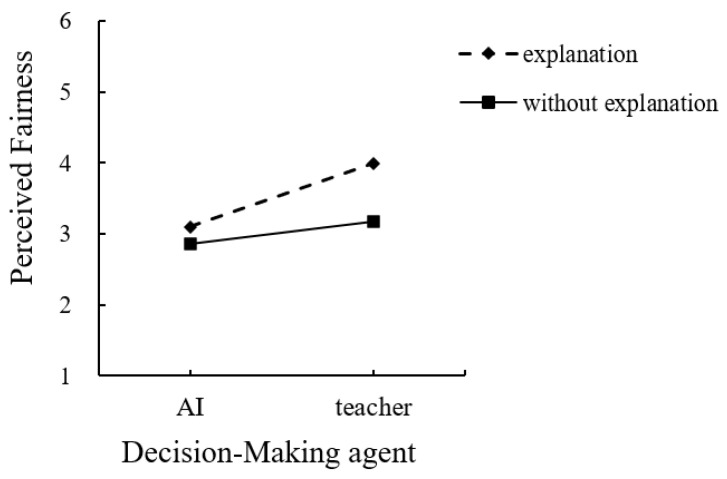
Moderating effect plot of explanatory feedback.

Simple slope analysis was performed, and a moderating effect plot was constructed (see Figure 3) to unpack the interaction between explanatory feedback and the decision-making agent. The results indicated that the positive predictive effect of the decision-making agent on decision acceptance intensified as explanatory feedback increased. Specifically, when providing explanatory feedback, teacher-led decision-making exerted a substantially stronger positive influence on decision acceptance relative to AI-led decision-making, accompanied by a prominent gap in acceptance scores between the two conditions. Although the positive association between the decision-making agent and decision acceptance remains statistically significant when no explanatory feedback is offered, its effect size is markedly smaller relative to the condition with explanatory feedback. Overall, these findings confirm that explanatory feedback positively moderates the pathway through which perceived fairness indirectly influences decision acceptance. Higher levels of explanatory feedback further amplify the incremental advantage that human teacher raters possess over AI raters in boosting students’ decision acceptance.

**Figure 4:**
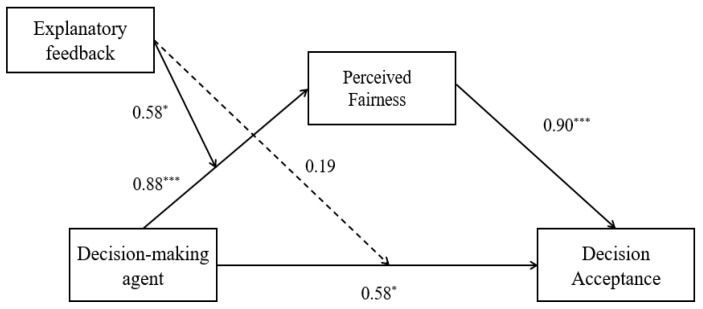
Moderated mediation path diagram of decision-making agent on decision acceptance. *, *** indicate significance at the 0.05 and 0.001 levels, respectively.

Taken together, the results supported the hypothesized moderated mediation model linking the decision-making agent, perceived fairness, explanatory feedback, and decision acceptance. Specifically, perceived fairness mediated the relationship between the decision-making agent and decision acceptance, whereas explanatory feedback only moderated the path from the decision-making agent to perceived fairness. Notably, the measurement of perceived fairness and decision acceptance relied on cross-sectional self-report data collected at a single time point, which limits causal inference regarding the directional relationship between the two constructs. While the experimental manipulation of the independent variable establishes its temporal priority, we cannot definitively confirm that perceived fairness causally shapes decision acceptance; reverse or reciprocal relationships between the two variables remain possible. The proposed moderated mediation model was empirically validated (see Figure 4).

## 4. Discussion

This study recruited junior high school students as participants and adopted a 2 (decision-making agent: teacher vs. AI) × 2 (explanatory feedback: explanation vs. without explanation) between-subjects experimental design. A moderated mediation model was constructed to systematically explore how a decision-making agent affects students’ decision acceptance in educational contexts, as well as the mediating role of perceived fairness and the moderating role of explanatory feedback. The results demonstrated that students exhibited significantly higher acceptance toward grading decisions made by teachers relative to AI. Perceived fairness partially mediated the association between the decision-making agent and decision acceptance, and explanatory feedback positively moderated only the path from the decision-making agent to perceived fairness, verifying the overall moderated mediation model. In addition, we acknowledge that the score of 6 out of 10 used in this study represents a relatively unfavorable outcome for students. Given that the favorability of evaluation results may substantially shape students’ perceived fairness, perceived trust, and decision acceptance, all findings derived from the current experiment are limited to contexts featuring disadvantageous scoring feedback. Future research could further explore divergent affective and attitudinal responses when participants receive favorable evaluation outcomes. The following discussion elaborates on these findings against relevant theoretical foundations.

### 4.1. Decision-Making Agent and Decision Acceptance

The current findings fully support Hypothesis 1, indicating that students demonstrate higher acceptance of decisions toward teacher-made evaluations compared with AI-generated decisions. This result aligns with preferences regarding human–AI decision-making and the empirical evidence documented in prior studies (Daschner & Obermaier, 2022; Wesche et al., 2024; Vanneste & Puranam, 2025). In educational evaluation contexts, teachers act as traditional authoritative figures. Long-term daily interactions build stable emotional connections and trust between teachers and students, making teacher grading judgments more humanized, context-adaptive and communicable in students’ eyes (Archambault et al., 2022). In contrast, AI decisions rely on standardized algorithmic rules and lack emotional empathy and situational flexibility. Students generally consider the assessment methods of artificial intelligence to be rigid and lacking in the human touch. The absence of explanatory information during the decision-making process exacerbates this feeling (Moreno, 2004), thereby reducing their acceptance of the decision. This study extends the application scope of human–machine preference to the context of junior high school assignment evaluation. It confirms that students still prefer human teacher decisions even in objective and standardized scoring tasks, highlighting the core value of emotional trust and humanistic attributes in educational contexts.

### 4.2. Mediating Role of Perceived Fairness

The results support Hypothesis 2. Perceived fairness significantly partially mediates the link between the decision-making agent and the acceptance of the decision. The mediated effect accounts for 67.52%. In other words, more than two-thirds of the effect comes from improved students’ perceived fairness. Based on organizational fairness theory, students do not accept decisions only based on final scores. Their feelings about procedural and interpersonal fairness matter more (Pitman, 2016). Most students regard teacher grading as fair: teachers follow unified rules while taking individual student differences into account, making grading logic easy to comprehend. In contrast, AI scoring rules lack transparent explanations; low algorithm transparency reduces students’ perceived fairness and further lowers decision acceptance.

This study identifies a complete psychological mechanism whereby the type of decision-making agent predicts perceived fairness, which in turn shapes students’ decision acceptance. It offers a clear mediation framework for human–AI evaluation in education. Earlier research focused mostly on technical performance. This work makes up for the gap by exploring inner psychological mechanisms.

### 4.3. Moderating Role of Explanatory Feedback

The findings partially support Hypothesis 3. Explanatory feedback positively moderates only the first path from the decision-making agent to perceived fairness. It has no significant moderating effect on the path from perceived fairness to acceptance of the decision. Simple effect analysis shows scoring explanations raise perceived fairness significantly under both AI and teacher conditions. The improvement is larger in the teacher group. This result matches Mitchell’s (2011) findings. Explanatory feedback is an effective tool that can enhance students’ acceptance of the results and improve their perception of fairness. For AI decisions, detailed explanations can reveal some of the reasons behind the decisions, helping students understand the scoring logic. For teacher decisions, extra explanations strengthen teachers’ credibility and further expand their advantage in boosting perceived fairness.

However, explanatory feedback did not significantly moderate the effect of the decision-making agent on acceptance of the decision. This indicates that the provision of explanations cannot alter the direct influence of the decision-making agent on the acceptance of the decision. Regardless of whether scoring explanations were provided, students’ acceptance of teacher decisions remained consistently and significantly higher than their acceptance of AI decisions. Explanatory feedback did not moderate this core difference. This divergent finding reconciles the conflicting conclusions in the existing literature. Choi and Chao (2024) claimed AI decisions are perceived as fairer under unfavorable outcomes among adult participants, yet the present study’s sample of junior high students yields opposite results. Such inconsistency can be attributed to adolescents’ unique teacher–student attachment and limited exposure to algorithmic evaluation, which override the “objective algorithm” stereotype observed in adult workplace samples. In addition, the provision of detailed explanations amplifies students’ ability to distinguish human empathy from rigid algorithmic logic, widening the fairness gap rather than bridging it.

Two potential reasons account for the non-significant moderating effect in the direct pathway. On the one hand, junior high school students engage in continuous and real classroom interactions with their teachers, which cultivates stable and reliable trust. Students are naturally willing to accept teacher grading results based on long-term trust and obedience, even without detailed scoring explanations. Providing explanations only further improves their acceptance, serving as a supplementary advantage rather than a fundamental change. In contrast, AI systems lack real interpersonal interaction. Students subjectively perceive algorithmic evaluation as cold and inhumane. A single scoring explanation is insufficient to eliminate long-standing psychological biases against AI. From the perspective of adolescent human–machine interaction psychology, explanatory feedback makes students explicitly perceive two distinct evaluation logics: teachers integrate personal care and situational context into scoring explanations, while AI only provides mechanical rule-based deductions (Chen et al., 2026). Transparent explanations highlight the humanistic advantages of teacher evaluation and the emotional deficiency of algorithmic scoring simultaneously. For teenagers who prioritize interpersonal communication in school life, such a clear contrast further enlarges their differential fairness perception of the two decision-making agents (Liao et al., 2024). Consequently, the acceptance of the decision for teacher-led evaluation remains consistently higher than for AI-led evaluation at all levels of explanatory feedback, and explanatory feedback intervention cannot eliminate this inherent emotional gap. On the other hand, most junior high school students remain unfamiliar with artificial intelligence technology. A single evaluation experience cannot reshape their stable cognitive attitudes toward AI. This suggests that the transformation of digital education acceptance requires continuous daily interaction with AI tools, so as to gradually reduce students’ inherent resistance and negative perceptions of algorithmic decision-making.

In summary, explanatory feedback works by altering how strongly the decision-making agent predicts perceived fairness. It does not directly moderate the link between the decision-making agent and the acceptance of the decision. This finding clarifies the specific functional path and boundary conditions of explanatory feedback in human–AI educational evaluation.

### 4.4. Theoretical Implications

Centered on students’ psychological perceptions in educational settings, this study directly compares student acceptance of teacher versus AI decisions. It expands the research scope of algorithmic decision-making from organizational management to basic education and broadens the research boundary of human–machine decision-making disparities. Furthermore, this study validates that perceived fairness acts as a core mediating variable linking the decision-making agent and acceptance of the decision. It reveals the underlying mediating mechanism and clarifies the internal psychological pathway that shapes students’ acceptance of educational decisions. Importantly, the current findings confirm that explanatory feedback only moderates the first-stage path of the model. This outcome clarifies the specific functional stage of explanatory feedback, improves the theoretical framework of moderated mediation in educational human–machine decision-making, and further refines the theoretical system of intelligent educational evaluation. This study’s conclusions are bounded by unfavorable evaluation outcomes (a score of 6/10). For scenarios with favorable high scores, the psychological gap between teacher and AI decision acceptance may shrink, as students’ positive affective experience will weaken the sensitivity to decision-makers’ identity. Future theoretical models should incorporate outcome favorability as an additional boundary condition to enrich the framework of human–AI educational decision acceptance.

### 4.5. Practical Implications

The present findings offer valuable practical insights for promoting educational digital transformation and optimizing human–machine collaborative decision-making. First, in daily educational scenarios such as homework correction, academic evaluation, and student behavior management, educators should adhere to the principle of “human–machine collaboration and teacher leadership”. AI should not fully replace teachers in educational decision-making. Schools should reserve teachers’ final decision-making authority and interpretation power. Relying on long-term emotional bonds and mutual trust between teachers and students can effectively maintain students’ acceptance of decisions and psychological recognition of educational evaluations.

Second, interpretability should be prioritized in the design of AI educational tools. Clear, specific and comprehensible grading criteria should be provided to students throughout the scoring, evaluation and feedback processes. Transparent decision-making logic can balance information fairness in the decision-making process, reduce the perceived fairness loss of students regarding AI decisions, and alleviate their rejection of algorithms.

Third, standardized explanation procedures should be matched with teacher-led evaluations. Whether teachers adopt AI-assisted tools or not, they should clearly state the reasons and basis for every evaluation result. This stable and consistent interpretive practice can continuously strengthen students’ perceived fairness and further improve their acceptance and cooperation with educational decisions.

Finally, schools should carry out systematic AI literacy education for junior high school students. Such training helps students understand the basic logic, operational rules, and applicable boundaries of AI scoring. It reduces students’ unfamiliarity, resistance, and distrust toward algorithm-based evaluation, thereby laying a solid cognitive and psychological foundation for the high-quality implementation of human–machine collaborative education.

### 4.6. Limitations and Future Research

This study preliminarily uncovers how a decision-making agent shapes students’ acceptance of decisions in educational settings, yet several limitations exist. First, participants were only grade seven junior high students from one city in Henan Province. The narrow sample limits representativeness, so findings should be generalized cautiously to students from other regions or schooling stages. Second, this research relies on scenario-based experiments. Such simulated settings differ from real-class homework grading and on-site feedback, lowering the ecological validity of the results. Third, only standardized math homework scoring is examined. Subjective and complicated educational decisions, including mental health assessment, reward and punishment management, and comprehensive quality evaluation, are excluded, restricting the generalizability of conclusions. Finally, we must admit that in the experiment, we set the teacher as the decision-making agent for the students, who were more familiar with the teacher. This might have led to the confounding of multiple interrelated variables, such as the long-term familiarity between the teacher and the students, stable interpersonal trust, the teacher’s accountability status within the school, local classroom authority, and the teacher’s exclusive subject-specific contextual knowledge. Therefore, although this design can compare the two types of real decision-making agents in the students’ school environment, it has good ecological validity, but it cannot separate the pure effect of “whether the decision-maker is a human or an AI” from the accompanying familiarity, relationship trust, and authority perception.

Accordingly, future research can be improved in four directions: first, recruit samples across different grades, regions and subjects to strengthen external validity; second, adopt longitudinal designs and collect real classroom evaluation data to boost ecological validity; third, add control variables like teacher–student bond and AI trust to enrich and refine the moderated mediation model of acceptance of the decision; and finally, future experiments may adopt more refined experimental designs to disentangle overlapping confounding variables. For instance, researchers could add an experimental group with unfamiliar human raters to independently manipulate interpersonal familiarity while keeping the decision agent as a human; alternatively, baseline student–teacher trust can be measured as a continuous covariate to partial out its confounding effect on attitudes toward algorithmic decision-making. Another viable approach involves establishing four experimental conditions comprising familiar regular teachers, unfamiliar human instructors, standalone unsupervised AI, and AI scoring under human teacher oversight, which enables researchers to separately parse the unique independent effects of the decision agent’s human identity, interpersonal closeness, and human oversight of algorithmic systems on students’ perceived fairness and decision acceptance.

## 5. Conclusions

Synthesizing the above empirical results, theoretical analysis and practical implications derived from the moderated mediation model, this paper summarizes three core conclusions corresponding to the three research hypotheses proposed in the introduction. By integrating authority acceptance theory and organizational justice theory, this study draws three core conclusions: (1) junior high school students show significantly higher acceptance of teacher-made decisions than AI-generated decisions; (2) perceived fairness plays a significant partial mediating role between the decision-making agent and decision acceptance, meaning decision source boosts decision acceptance by elevating students’ perceived fairness; and (3) explanatory feedback positively moderates the path from the decision-making agent to perceived fairness. Specifically, providing students with rationales and explanations for scoring results further and effectively elevates their perceived fairness when the decision-making agent is a teacher.

In practice, educational digital transformation should follow core principles of human–machine collaboration, teacher leadership and transparent interpretation. While utilizing AI’s strengths in efficiency, standardization and traceability, educators need to value students’ emotional needs, feelings of fairness, and psychological experience. These efforts help advance human-centered, acceptable and high-quality digital education.

## Figures and Tables

**Table 1:** Descriptive statistics and correlation coefficients among study variables.

	*M*	*SD*	1	2	3	4
1. Decision acceptance	3.97	1.42	1			
2. Decision-making agent	0.50	0.50	0.35 **	1		
3. Perceived fairness	3.28	1.01	0.72 **	0.29 **	1	
4. Explanatory feedback	0.49	0.50	0.31 **	0.01	0.26 **	1

Note: AI = 0, teacher = 1; explanation = 1, without explanation = 0; *N* = 250; ** *p* < 0.01.

**Table 2:** Descriptive statistics of decision acceptance and perceived fairness by decision-making agent (N = 250).

Explanatory Feedback	Decision-Making Agent	Decision Acceptance	Perceived Fairness	*N*
Explanation	AI	3.83 (1.27)	3.11 (0.92)	64
	Teacher	5.01 (1.28)	3.99 (0.79)	63
Without explanation	AI	3.10 (0.88)	2.86 (0.92)	61
	Teacher	3.95 (1.50)	3.17 (1.03)	62

Note: Values represent means and standard deviations. AI = artificial intelligence.

**Table 3:** Moderated mediation analysis of decision-making agent on decision acceptance.

Result Variable	Prediction Variable	*R* ^2^	*F*	*β*	*t*	95% CI
Perceived fairness		0.17	13.27 ***			
	Decision-making agent			0.88	5.38 ***	[0.56,1.21]
	Explanatory feedback			0.24	1.43	[−0.08, 0.56]
	AI familiarity			0.04	0.66	[−0.09, 0.19]
	Frequency of AI usage			−0.01	−0.17	[−0.12, 0.10]
	X × W			0.58	2.45 *	[0.11, 1.03]
Decision acceptance		0.57	51.60 ***			
	Decision-making agent			0.58	3.24 **	[0.24, 0.95]
	Perceived fairness			0.90	13.74 ***	[0.77, 1.02]
	Explanatory feedback			0.52	3.07 **	[0.19, 0.86]
	AI familiarity			−0.06	−0.89	[−0.21, 0.08]
	Frequency of AI usage			0.07	−1.05	[−0.04, 0.19]
	X × W			0.19	0.77	[−0.29, 0.66]

Note: * *p* < 0.05, ** *p* < 0.01, *** *p* < 0.001. X × W mean decision-making agent × explanatory feedback. AI = artificial intelligence.

**Table 4:** The direct effect and the mediating effect of explanatory feedback.

	Explanatory Feedback	Effect	SE	Boot LLCI	Boot ULCI
Direct effect	Explanation	0.38 *	0.17	0.30	0.73
	Without explanation	0.57 ***	0.17	0.23	0.90
Indirect effect	Explanation	0.79 ***	0.14	0.52	1.09
	Without explanation	0.27	0.16	−0.03	0.59

Note: Direct effect path: decision-making agent → acceptance of decisions. Indirect effect: decision-making agent → perception of fairness → acceptance of decisions. * *p* < 0.05, *** *p* < 0.001.

## Data Availability

The data presented in this study are available on request from the corresponding author.

## Appendix A

### Appendix A.1. Fairness Perception Items

The teacher (or AI system) is fair and objective.	1	2	3	4	5
I think the score assigned to me by the teacher (or AI system) is fair.	1	2	3	4	5

### Appendix A.2. Decision Acceptance Items

I accept the grading result provided by the teacher (or AI system).	1	2	3	4	5	6	7
I believe the grading results are accurate.	1	2	3	4	5	6	7
I think the information provided by the teacher (or AI system) in the above scenario is precise.	1	2	3	4	5	6	7
If similar assignments are assigned in the future, I am willing to let the teacher (or AI system) grade my work again.	1	2	3	4	5	6	7
